# Supplementary material for: Diversity of Studies on Neighborhood Greenspace and Brain Health by Racialized/Ethnic Group and Geographic Region: A Rapid Review
Source: Int J Environ Res Public Health. 2023 Apr 27;20(9):5666. doi: 10.3390/ijerph20095666 (PMC10178609; doi:10.3390/ijerph20095666)
Supplement: Supplementary file 1 [file ijerph-20-05666-s001.zip › ijerph-2290990-supplementary.pdf]

Table S1. Characteristics of the 57 published studies of greenspace-ADRD brain health associations

Citation	Cohort/data source	Sample size	Population-based/ random sample	Geographic location	Age groups included	Racialized/ethnic groups included
Aitken (2021)	Medicare beneficiaries in Miami-Dade County	249,405	Yes	US	≥65 years	Hispanic: 66%<br>Black: 11%<br>NH White: 23%
Almeida (2022)	Generation XXI birth cohort	3,827	Yes	Portugal	<18 years	NA
Anabitarte (2022)	INMA cohort study (INfancia y Medio Ambiente (Environment and Childhood Project)	751	Yes	Spain	<18 years	NA
Asta (2021)	Gene and Environment Prospective Study on Infancy in Italy (GAS-PII)	465	Yes	Italy	<18 years	NA
Astell-Burt (2020a-HealthPlace)	Sax Institute 45 and up study	45,644	Yes	Australia	45-64 years, ≥65 years	NA
Astell-Burt (2020b-EnvirInt)	Sax Institute 45 and up study	109,688	Yes	Australia	45-64 years, ≥65 years	NA
Bagheri (2021)	Recruited from family physician practices	25,511	No	Australia	≥65 years	NA
Besser (2020)	Multi-Ethnic Study of Atherosclerosis (MESA)	4,084	Yes	US	45-64 years<br>≥65 years	Black: 27%<br>Chinese: 11%<br>Hispanic: 21%<br>White: 40%
Besser (2021a)	Multi-Ethnic Study of Atherosclerosis (MESA)	1,733	Yes	US	45-64 years<br>≥65 years	Chinese: 11%<br>African American: 29%<br>Hispanic: 17%<br>White: 43%
Besser (2021b)	Cardiovascular Health Study (CHS)	1,125	Yes	US	≥65 years	White: 89%<br>Black: 11%<br>Other: 0.3%
Bijnens (2020)	East Flanders Prospective Twin Survey (EFPTS)	620	Yes	Belgium	<18 years	NA

Bijnens (2022)	Flemish Environmental Health Surveys	596	No	Belgium	<18 years	NA
Binter (2022)	Human Early Life Exposome Project (HELIX)	5,403	Yes	UK, France, Spain, Greece	<18 years	NA
Brown (2018)	Medicare beneficiaries in Miami-Dade County	249,405	Yes	US	≥65 years	Hispanic: 66%<br>NH White: 23%<br>Black: 11%
Cerin (2021)	Australian Diabetes, Obesity and Lifestyle (AusDiab) study	4,141	Yes	Australia	18-44 years, 45-64 years, ≥65 years	NA
Cherrie (2018)	Lothian birth cohort 1936	281	Yes	UK	18-44 years, 45-64 years, ≥65 years	NA
Cherrie (2019)	Lothian birth Cohort 1936	281	Yes	UK	≥65 years	NA
Clarke (2012)	Chicago Community Adult Health Study	949	Yes	US	45-64 years, ≥65 years	White: 43%<br>Black: 37<br>Hispanic: 18%<br>Other: 3%
Crous-Bou (2020)	ALFA (Alzheimer and Families study)	958	No	Spain	45-64 years, ≥65 years	NA
Dadvand (2015)	Barcelona primary schools	2,593	No	Spain	<18 years	NA
Dadvand (2017)	Sabadell and Valencia (INfancia y Medio Ambiente (INMA) cohorts	987	Yes	Spain	<18 years	NA
Dadvand (2018)	Brain Development and Air Pollution Ultrafine Particles in School Children (BREATHE)	253	No	Spain	< 18 years	NA
De Keijzer (2018)	Whitehall II cohort	6,506	Yes	UK	45-64 years, ≥65 years	White: 91%<br>Other: 9%
Dockx (2022)	ENVIRONAGE birth cohort	456	Yes	Belgium	<18 years	NA
Dzhambov (2019)	Convenience sample	112	No	Bulgaria	45-64 years	NA
Falcon (2021)	Alzheimer and Families cohort (ALFA)	212	No	Spain	45-64 years, ≥65 years	NA

FangFang (2022)	Anhui Healthy Longevity Survey (AHLS)	5,848	Unclear	China	45-64 years, ≥65 years	NA
Finlay (2021)	REasons for Geographic and Racial Differences in Stroke (REGARDS) and semi-structured interview/qualitative sample	REGARDS: 21,151<br><br>Semi structured interviews: 125	Yes (REGARDS)<br><br>No (qualitative sample)	US	45-64 years, ≥65 years	REGARDS: Black: 40% White: 60%<br><br>Qualitative sample: White: 57% Black 25% Other 18%
Flouri (2019)	UK Millennium cohort	4,758	Yes	UK	<18 years	White: 74% Other: 36%
Hystad (2019)	CARTaGENE cohort	6,658	Yes	Canada	18-44 years, 45-64 years, ≥65 years	White: 81% Other: 19%
Jimenez (2021)	Project Viva (prebirth cohort)	857	No	US	<18 years	White: 71% Non-White: 29%
Jin (2021)	Chinese Longitudinal Healthy Longevity Survey	1,199	Yes	China	≥65 years	Han: 90% Minority ethnicity: 10%
Ju (2021)	Korea Community Health Survey	191,054	Yes	Korea	18-44 years, 45-64 years, ≥65 years	NA
Julvez (2021)	Human Early-Life eXposome project (HELIX):<br><br>BiB (Bradford, UK), EDEN (France), INMA (Spain), KANC (Lithuania), MoBA (Norway) Rhea (Greece)	1,298	Yes	UK, France, Spain, Lithuania, Norway, Greece	<18 years	NA
Kuhn (2017)	Berlin Aging Study II	341	No	Germany	45-64 years, ≥65 years	NA
Lee (2021)	Environment and Development of Children cohort study	189	No	Korea	<18 years	NA

Lega (2021)	Convenience sample	185	No	UK	18-44 years, 45-64 years, ≥65 years	NA
Liao (2019)	Birth cohort in Wuhan, China	1,312	No	China	<18 years	NA
Liu (2019)	National Health Insurance Research Databases (NHIRD) (cases), Longitudinal Health Insurance database of NHIRD (controls)	24,802	Yes	Taiwan	≥65 years	NA
Liu (2020)	National Health Insurance Research Databases (NHIRD) (cases), Longitudinal Health Insurance database of NHIRD (controls)	52,412	Yes	Taiwan	≥65 years	NA
Maes (2021)	SCAMP (Study of cognition, adolescents, and mobile phones)	3,568	No	UK	<18 years	White: 45% Black: 15% Asian: 27% Mixed: 11% Other: 1%
Paul (2020)	Ontario Population Health and Environment Cohort (ONPHEC)	1.74 million dementia cohort, 4.25 million stroke cohort	Yes	Canada	18-44 years, 45-64 years, ≥65 years	NA
Reuben (2019)	Environmental Risk (E-Risk) Longitudinal Twin Study	1,658	Yes	UK	<18 years	NA
Slawsky (2022)	Gingko Evaluation of Memory Study (GEMS)	3,047	Yes	US	≥65 years	White: 95% Other: 5%
Sylvers (2022)	REasons for Geographic and Racial Differences in Stroke (REGARDS)	10,289	Yes	US	45-64 years, ≥65 years	Black: 31% White: 69%
Tani (2021)	Japan Gerontological Evaluation Study	76,053	Yes	Japan	≥65 years	NA

Wang (2017)	Community-based cohort	3,544	No	China	≥65 years	NA
Ward (2016)	Schools in Auckland	72	No	New Zealand	<18 years	NA
Wu (2015)	Cognitive Function and Ageing Study (CFAS)	2,424	No	UK	≥65 years	NA
Wu (2017)	Cognitive Function and Ageing Study II (CFAS)	7,505	No	UK	≥65 years	NA
Wu (2020)	Cognitive Function and Ageing Study II (CFAS), 10/66 study	CFAS: 4,955<br><br>10/66: 3,386	Yes	UK<br>China, Dominican Republic, Mexico	≥65 years	NA
Wu (2021)	Center for Medicaid and Medicare (CMS) dataset	~106,763	Yes	US	≥65 years	Black: 9%<br>White: 86%<br>Other: 5%
Yu (2018)	Cohort study on osteoporosis and general health (Mr. and Mrs. Os)	3,240	No	≥65 years	≥65 years	NA
Yuchi (2020)	Medical Services Plan Physician Visit and Hospital discharge data	678,000	Yes	British Columbia, Canada	45-64 years, ≥65 years	NA<br>(Note: indicated whether participant lived in neighborhood with higher concentration of Chinese or South Asian residents)
Zhu (2019)	Chinese Longitudinal Healthy Longevity Survey	38,327	Yes	China	≥65 years	Han - 94.3%<br>Other – 5.7%
Zhu (2020)	Chinese Longitudinal Healthy Longevity Survey	6,994	Yes	China	≥65 years	NA
Zijlema (2017)	Positive health effects of natural outdoor environment in typical populations in different regions in Europe (PHENOTYPE)	1,628	Yes	Spain, Netherlands, UK	18-44 years, 45-64 years, ≥65 years	NA
NA = Not applicable (in main Table 1, we assigned racialized/ethnic group by assuming relatively homogenous population in the country where the sample originated)						

* Unclear if population-based from paper

Table S2. Greenspace and ADRD brain health measures in the 57 published studies of greenspace-ADRD brain health associations

**Citation**	**Greenspace measure(s)** (O=objective; S=self-reported)	**GIS buffer(s) for greenspace measure** (NA=not applicable; R=residential, S=school, C=commute)	**Greenspace measured as change over time** (alternative is single time points such as baseline visit or average over visits)	**ADRD brain health outcome(s)**	**ADRD outcome measured as change over time** (e.g., longitudinal change in cognition) (alternative is one time point or multiple time points included in model)
Aitken (2021)	Normalized difference vegetation index (NDVI) (O)	US census block (R)	No	AD diagnosis,<br>ADRD diagnosis (including AD),<br>Non-AD dementia diagnosis	No
Almeida (2022)	Normalized difference vegetation index (NDVI) (O),<br>Availability of urban green space (O),<br>Number of urban green spaces (O),<br>Distance to nearest green space (O)	Circular buffer (R, S)	No	Wechsler Intelligence Scale for children (WISC-III):<br>Verbal IQ,<br>Performance IQ,<br>Global IQ	No
Anabitarte (2022)	Normalized difference vegetation index (NDVI) (O),<br>Vegetation continuous field (VCF) (O),<br>Access to greenspace (O),<br>Distance from home to greenspace (O)	Circular buffer (R)	No	Attention network test (ANT) (attention):<br>Hit Rt median (median response time)<br>HRT-SE (standard error of the response time)	Yes
Asta (2021)	Normalized difference vegetation index (NDVI) (O)	Circular buffer (R)	No	IQ:<br>Total IQ<br>Verbal IQ<br>Performance IQ<br><br>Composites:<br>Verbal comprehensive index<br>Perceptual Organization index<br>Freedom from distractibility index	No

				Processing speed index<br><br>Subtests:<br>Information, similarities, comprehension, arithmetic, digit span, picture completion, picture arrangement, block design, object assembly, mazes, coding, symbol search	
Astell-Burt (2020a-HealthPlace)	Created an objective latent class variable of green space groups (n=8) that each comprised a different % of tree canopy, open grass, and shrub combinations (O)	Road network buffer (R)	No	Self-rated memory:<br>Excellent memory<br>Subjective complaints<br>Incident excellent memory<br>Incident subjective complaint	Yes
Astell-Burt (2020b-EnvirInt)	Total greenspace (%) (O)<br>Tree canopy (%) (O)<br>Open grass (%) (O)	Road network buffer (R)	No	Incident dementia:<br><br>First anti-dementia medication prescription<br><br>First hospitalization or death with dementia reported<br><br>Combined measure	Yes
Bagheri (2021)	Area of public open space (O)	Statistical area level 1 (SA1) (R)	No	Predicted risk of dementia, Diagnosis of dementia	No
Besser (2020)	% Park space (O)	Circular buffer (R)	No	Cognitive measures:<br>Cognitive Abilities Screening Instrument (CASI) (global cognition),<br>Digit Span Forward and Backward (DSF, DSB) (memory),<br>Digit Symbol (DS) (processing speed)	No
Besser (2021a)	% Park space (O)<br>Distance to nearest park (O)	Circular buffer (R)	No	Cognitive measures:<br>Cognitive Abilities Screening Instrument (CASI) (global cognition),<br>Digit Symbol (DSC) (processing speed)	Yes

Besser (2021b)	% total greenspace (O)	Circular buffer (R)	No	MRI outcomes:<br>Left hippocampus<br>Right hippocampus<br>White matter grade<br>Ventricle grade	No
Bijnens (2020)	Percentage greenspace (seminatural, forested, blue, and urban green) (O),<br>High green (vegetation height >3m) (O)	Circular buffer (R)	No	Intelligence (WISC-R):<br>Verbal IQ,<br>Performance IQ,<br>Total IQ	No
Bijnens (2022)	Green space index for high and low green vegetation (based on averaging residential and school greenspace weighted by time expected to spend in those places per day) (O),<br>Access to greenspace based on area size and maximum distance to green (in neighborhood, green in district, small urban green, urban green, urban forest) (O)	Circular buffer (R)	No	Stroop test (selective attention),<br>Continuous Performance test (sustained and selective attention),<br>Digit span (short term memory),<br>Digit symbol test and Pattern Comparison test (visual information processing speed)	No
Binter (2022)	Normalized difference vegetation index (NDVI) (O)<br><br>Residential proximity to major natural spaces (green or blue spaces) (O)	Circular buffer (R)	No	(Different tests of verbal and non-verbal abilities depending on cohort):<br>British Picture Vocab Scale,<br>Weschler preschool and primary scale of intelligence,<br>McCarthy Scales of Children's abilities	No
Brown (2018)	Normalized difference vegetation index (NDVI) (O)	US Census block (R)	No	AD diagnosis	No
Cerin (2021)	% Park land (O)	Street network buffer (R)	No	CA verbal learning test (CVLT) (memory),	No

				Symbol-digit modalities test (processing speed)	
Cherrie (2018)	Percentage of park area (O)	Circular buffer (R)	No	Moray House Test N. 12 (MHT) (intelligence)	Yes
Cherrie (2019)	Percentage of park area (O)	Circular buffer (R, S, C)	No	Moray House Test N. 12 (MHT) (intelligence)	Yes
Clarke (2012)	Park area in square miles (O)	US Census tract (R)	No	Modified Telephone Instrument for Cognitive Status (TICS) (global cognition)	No
Crous-Bou (2020)	Normalized difference vegetation index (NDVI) (O)	Circular buffer (R)	No	Cognition Composite Score (Memory Binding Test (MBT) immediate total paired recall, MBT delayed free recall, WAIS-IV coding, semantic fluency)<br><br>Composite-global episodic memory (from MBT and WAIS-IV subtests)<br><br>Composite-executive function (from MBT and WAIS-IV subtests)<br><br>MRI: AD regions of interest cortical thickness; ventricular volume; hippocampal volume	No
Dadvand (2015)	Normalized difference vegetation index (NDVI) (O) (time-weighted average of proximity to greenness by buffer type)	Circular buffer (R), Circular buffer (C), Circular buffer (S)	No	Computerized N-back testing (working memory), Computerized attentional network test (ANT) (attention)	Yes
Dadvand (2017)	Normalized difference vegetation index (NDVI) (O), Vegetation Continuous Fields (VCF) (O)	Circular buffer (R)	No	Conners' Kiddie Continuous Performance Test (K-CPT) (attention), Attentional Network Test (ANT) (attention)	Yes
Dadvand (2018)	Normalized difference vegetation index (NDVI) (O) (averaged around all home addresses since birth)	Circular buffer (R)	No	Brain volumes from magnetic resonance imaging (3D MRI)	No

De Keijzer (2018)	Normalized difference vegetation index (NDVI) (O), Enhanced Vegetation Index (EVI) (O)	Postcode centroid (R)	No	Alice Heim 4 test (reasoning), "S words and animal names" (phonemic and semantic verbal fluency), Free recall test (Short term memory), Global cognition z-score	Yes
Dockx (2022)	Non-agricultural vegetation from land cover data (O)	Circular buffer (R)	No	CANTAB (Cambridge Neuropsychological Test automated battery):<br><br>Motor screening task and Big/Little circle (attention and psychomotor speed)<br><br>Spatial Span test and delayed matching sample (visual recognition/working memory)	No
Dzhambov (2019)	Normalized difference vegetation index (NDVI) (O)	Circular buffer (R)	No	Consortium to Establish Registry for Alzheimer's disease neuropsychological battery (CERAD-NB):<br>Verbal fluency (fluency)<br>Modified Boston Naming Test (naming),<br>Word List Memory (memory),<br>Word List Recall (memory),<br>Word List Recognition,<br>Montreal cognitive assessment (MoCA),<br>MRI of cortical thickness of multiple brain regions of interest	No
Falcon (2021)	Normalized difference vegetation index (NDVI) (O)	Circular buffer (R)	No	MRI:<br>Gray matter volume (including regions of interest),<br>white matter volume (including regions of interest)	No
FangFang (2022)	Distance from home to nearest park (O),<br>% green space (O)	Circular buffer (R)	No	Mini Mental State Exam (MMSE) score,<br>Presence of mild cognitive impairment (MCI)	No
Finlay (2021)	Guiding semi-structure interview question:	NA	No	One composite measure created from:	No

	"How and where did participants exercise out of their home" (S)<br><br>Number of parks (O)	Circular buffer (R)		Animal fluency test and letter fluency test (language/executive function),<br>World list learning (verbal learning),<br>World list delayed (verbal memory),<br>MoCA subset (verbal memory and orientation)	(change in score over time examined in supplemental analysis, but did not report/provides estimates for park variable)
Flouri (2019)	Percentage greenspace (O)	Ward (R)	No	Cambridge Neuropsychological test Automated battery (CANTAB):<br>Spatial working memory	No
Hystad (2019)	Normalized difference vegetation index (NDVI) (O)	Circular buffers around postal code (R)	Yes	Computerized tests for:<br>Reaction time test,<br>Paired associates learning (working memory),<br>Verbal and numeric reasoning (executive function)	No
Jimenez (2021)	Normalized difference vegetation index (NDVI) (O)	Circular buffer (R)	No	Early childhood:<br>Peabody Picture vocabulary test (vocab comprehension),<br>Visual motor subtest of Wide Range Assessment of Visual Motor Abilities (WRAVMA) (visual-motor, fine motor, visuospatial skills)<br><br>Mid childhood:<br>Kaufman Brief Intelligence test (crystallized and fluid intelligence),<br>Visual motor subtest of WRAVMA (visual-motor, fine motor, visuospatial skills),<br>Visual Memory index of Wide Range Assessment of Memory and Learning (memory and learning)	No
Jin (2021)	Normalized difference vegetation index (NDVI) (O)	Circular buffer (R)	No	Cognitive impairment (Mini Mental State Exam (MMSE)<25),<br>MMSE score	No
Ju (2021)	Rating on "satisfaction in surrounding green spaces such as park and trees along streets" (S)	NA	No	Subjective cognitive decline from BRFSS module ("During past 12 months, have you experienced confusion or memory loss that is	No

				happening more often or is getting worse?")	
Julvez (2021)	Normalized difference vegetation index (NDVI) (O), Presence of major greenspace (O)	Circular buffer (R, S)	No	3 cognitive domains: Raven Coloured Progressive Matrices Test (fluid intelligence), Attention Network Test (attention), N-Back test (working memory)	No
Kuhn (2017)	Amount of forest and urban green (O)	Circular buffer (R)	No	Magnetic Resonance Imaging (CS) of integrity of amygdala, pregunual anterior cingulate cortex (pACC), and dorsolateral prefrontal cortext (DLPFC) determined from indicators of brain structural integrity (grey matter volume, magnetization transfer ratio, mean diffusivity)	No
Lee (2021)	Residential greenness (natural and built) (O)	Circular buffer (R)	No	Korean Educational Developmental Institute's Wechsler Intelligence Scale for Children: Verbal IQ, Performance IQ	No
Lega (2021)	Normalized difference vegetation index (NDVI) (O)	Buffer (R)	No	Forward Digit Span (short-term memory), Backward Digit Span (working memory), FDS+BDS=total memory	No
Liao (2019)	Normalized difference vegetation index (NDVI) (O)	Circular buffer (R)	No	Bayley Scales of Infant Development (BSID): Mental development index (MDI) (perceptual acuities, memory, learning and problem solving, abstract thinking)	No
Liu (2019)	Density of parks, greenery, and space as proxy for availability of green environment (O)	Township (R)	No	Incident AD dementia diagnosis	Yes
Liu (2020)	Density of parks, greenery, and space as proxy for availability of green environment (O)	Township (R)	No	Incident dementia diagnosis	Yes

Maes (2021)	Urban natural environments: 1) natural space, green versus blue space, grassland versus woodland<br><br>Constructed Daily exposure rate for: 1) natural space, 2) green versus blue; and 3) woodland versus grassland, based on predicted time spent at home and school.	Circular buffer (R,S)	No	Composite score of three computerized executive function tasks:<br>Backwards digit span,<br>Spatial working memory,<br>Trail Making task	Yes
Paul (2020)	Normalized difference vegetation index (NDVI) (O)	Circular buffer around home (R)	No	Incident case of dementia	Yes
Reuben (2019)	Normalized difference vegetation index (NDVI) (O)	Circular buffer (R)	No	Wechsler Intelligence Scale:<br>Fluid intelligence,<br>Crystalized intellectual performance<br><br>Cambridge Neuropsychological Test Automated Battery:<br>Executive function,<br>Working memory,<br>Attention	Yes
Slawsky (2022)	Composite greenspace metric based on: NDVI, % park space, distance to nearest park (O)	Circular buffer (R)	No	Incident all cause dementia,<br>Incident AD dementia,<br>Incident mixed/vascular dementia	Yes
Sylvers (2022)	Lack of parks/playgrounds in the neighborhood (S)<br><br>Number of neighborhood parks (O)	NA (R)<br><br>US Census tract (R)	No	CERAD World List learning (episodic memory)<br><br>Word List Delayed Recall (Episodic memory)<br><br>Animal Fluency Test (semantic memory and exec function)	No

				Letter fluency<br><br>Delayed recall on NINDS-CSN neuropsychological battery<br><br>Created overall cognitive status using factor scores from 5 tests<br><br>In sensitivity analysis, used Six Item Screener (SIS) to develop measure of cognitive impairment (based on cognitive test)	
Tani (2021)	Number of parks (O)	Elementary school district (S)	No	Dementia diagnosis/incidence	Yes
Wang (2017)	Normalized difference vegetation index (NDVI) (O)	Circular buffer (R)	No	Mini Mental State Exam (MMSE)	No
Ward (2016)	Time spent in green space (parks, sports fields, reserves) (O)	NA	No	CNS Vital Signs (CNS-VS): Visual memory, verbal memory, processing speed, psychomotor speed, reaction time, cognitive flexibility, executive function	No
Wu (2015)	Percentage of greenspace/private gardens (O)	Lower-layer super output area (LSOA) (R)	No	Cognitive function:<br>Cognitive impairment (Mini-Mental State Examination MMSE≤25)<br>Dementia:<br>Geriatric Mental Status and Automatic Geriatric Examination for Computer Assisting Taxonomy (GMS)	No
Wu (2017)	Percentage of greenspace/private gardens (O)	Lower-layer super output area (LSOA) (R)	No	Cognitive function:<br>Cognitive impairment (Mini-Mental State Examination MMSE≤25)<br>Dementia:<br>Geriatric Mental Status and Automatic Geriatric Examination for Computer Assisting Taxonomy (GMS)	No
Wu (2020)	Distance to park (O), % local green space (O),<br>% nature (O)	Circular buffer (R)	No	Dementia determined from:<br>Geriatric Mental State examination (GMS)	No

Wu (2021)	% greenspace (trees and herbaceous from land cover dataset) (O)	Zip code (R)	No	Risk of AD dementia diagnosis	Yes
Yu (2018)	Normalized difference vegetation index (NDVI) (O)	Circular buffer (R)	No	Cognitive Function: Mini-Mental state examination	No
Yuchi (2020)	Normalized difference vegetation index (NDVI) (O)	Circular buffer (R)	No	Dementia:<br>Non-AD dementia<br>AD<br>Parkinson's disease	Yes
Zhu (2019)	Normalized difference vegetation index (NDVI) (O)	Circular buffer (R)	Yes	Mini Mental State Examination (MMSE):<br><br>MMSE Score<br>Cognitive impairment (MMSE <24)	Yes
Zhu (2020)	Normalized difference vegetation index (NDVI) (O)	Circular buffer (R)	No	Mini Mental State Examination (MMSE<24)	No
Zijlema (2017)	Residential distance to natural outdoor environment (O), Normalized difference vegetation index (NDVI) (O), Amount of natural outdoor environment (S); visits to natural outdoor environment (S); time visiting natural outdoor environment (S)	Circular buffer (R)<br><br>Self-reported neighborhood	No	Cognitive function:<br>Color Trails Test (time and errors) (visual attention/effortful executive processing)	No

Table S3. Greenspace and ADRD brain health measures in the 57 published studies of greenspace-ADRD brain health associations

**Citation**	**Statistical methods, Covariates**	**Association between greenspace and ADRD outcome**	**Examined differences in associations by race/ethnicity?** (Method, if applicable)	**Associations by race/ethnicity** (if applicable)	**Health disparities or related framework** (e.g., social/structural determinants of health) referenced in paper
Aitken (2021)	Hierarchical/multilevel logistic regression analyses<br><br>Covariates: age, gender, racialized/ethnic group, median household income, number of comorbidities)	Compared to low greenness/NDVI, those living in high NDVI had lower odds of AD.<br><br>Compared to low greenness/NDVI, those living in medium and high NDVI had lower odds of ADRD.<br><br>No associations between NDVI and non-AD dementia.	Yes (interaction term testing between NDVI and racialized/ethnic group)	No interaction indicated between NDVI and racialized/ethnic group (Non-Hispanic White, Hispanic, Black)	No
Almeida (2022)	Hierarchical/multilevel linear mixed model<br><br>Covariates: sex, maternal education, monthly household income, population density, neighborhood deprivation index, level of urbanicity, school type	If urban green space available around home, better performance IQ and global IQ.<br><br>Greater number of greenspaces around home not associated with cognition.<br><br>Greater distance from home or school to greenspace not associated with cognition.<br><br>Greater greenness/NDVI around home not associated with cognition but around school associated with worse verbal, performance, and global IQ.<br><br>Greenspace around school	No (NA)	NA	No

		(presence and number) not associated with cognition.			
Anabitarte (2022)	General linear models (cross-sectional analysis)<br><br>General linear mixed-effects models (longitudinal analysis)<br><br>Covariates: SES, age at attention test, sex, preterm maternal IQ, maternal smoking during pregnancy, cohort (random effect for subject in longitudinal analysis)	Cross-sectional association between greenness/NDVI and attention (HRT-SE score) at 11-13 years of age.<br><br>No other associations.	No (NA)	NA	No<br><br>(However, discussed vulnerable groups (older adults, children, those with chronic disease, and low SES) as more vulnerable to noise, extreme temperatures, and air pollution.)
Asta (2021)	Multiple linear regression models<br><br>Covariates: age at test, gender, maternal and paternal education, socioeconomic position at birth, maternal age at delivery, maternal smoking during pregnancy, number older siblings, psychologist administered tests	Greater residential NDVI/ greenness associated with better Arithmetic subtest score.<br><br>No other associations.	No (NA)	NA	No<br><br>(However, discussed children as vulnerable groups due to environment exposures during development.)
Astell-Burt (2020a-HealthPlace)	Multilevel logistic regression using Markov Chain Monte Carlo method<br><br>Covariates: age, gender, lived with another person, highest education, annual household	Those with more tree canopy (compared to open grass) had lower odds of subjective complaints and higher odds of self-rated excellent memory at baseline.	No (NA)	NA	No

	income, economic status (employed)				
Astell-Burt (2020b-EnvirInt)	Multilevel discrete time-to-event history modeling using Markov Chain Monte Carlo method<br><br>Covariates: age, gender, couple status, highest education, annual household income, economic status (employed), and area level SES additionally adjusted for in separate model	Higher risk of dementia medications with higher total greenspace and higher total tree canopy. Lower risk of dementia medications with less open grass.<br><br>Lower odds of dementia event (hospitalization or death) if greater tree canopy.<br><br>Additionally controlling for area SES removed some associations, but generally in same directions.	No (NA)	NA	No
Bagheri (2021)	Multilevel linear and logistic regression (depending on outcome)	No association between area of public open space and odds of diagnosed dementia. Greater area of public open space associated with reduced predicted risk of dementia.	No (NA)	NA	No
Besser (2020)	Multilevel linear regression<br><br>Covariate: age, sex, education, income, racialized/ethnic group, neigh SES, site, neighborhood population density, apolipoprotein E (APOE) e4, perceived safety walking day or night and crime, arthritis, cardiovascular and cerebrovascular disease, diabetes, depression, total minutes walking/week	Greater park space around home associated with better processing speed but associations varied and were positive, negative, or null depending on geographic location/site and cognitive outcome.	No (NA)	NA	No

Besser (2021a-MESA)	Multivariable random intercept logistic regression<br><br>Covariates: age, sex, education, racialized/ethnic group, income, apolipoprotein E (APOE) e4, number of moves, neighborhood SES, neighborhood population density, site, neighborhood safety, walking and crime, arthritis, cardiovascular and cerebrovascular disease, and diabetes	Greater percentage park space around home associated with maintained/improved global cognition (CASI).<br><br>No associations between percentage park space and other cognitive measures.<br><br>No association between distance to nearest park and cognitive measures.	Yes (stratified models by racialized group and used interaction terms to test for differences in associations by racialized group)	Among Black participants, neighborhood percentage park space borderline associated with maintained/improve d global cognition (versus declining over time). No association between park space and global cognition found for Whites.	No
Besser (2021b-CHS)	Linear regression (sensitivity analysis used linear regression with generalized estimating equations clustering on tract)<br><br>Covariates: age, sex, racialized/ethnic group, income, education, neighborhood median household income, whole brain volume, site, comorbidities (hypertension, arthritis, diabetes, cardiovascular and cerebrovascular disease), pack years smoked, apolipoprotein E (APOE) e4	Borderline association between greater percentage greenspace and lower ventricle grade score.<br><br>No associations observed between greenspace and other imaging outcomes.	No (NA)	NA	No
Bijnens (2020)	Multilevel linear regression	Greater greenspace in pregnancy and in childhood was associated with higher	No (NA)	NA	No

	Covariates:<br>Childhood model: age, parental education, neighorhood household income, year of intelligence test, zygosity, chorionicity<br><br>Pregnancy model: also controlled for birth weight, gestational age, birth year, cord insertion, and maternal smoking during pregnancy	total IQ, higher Verbal IQ and higher Performance IQ for those children living in urban areas.<br><br>No association for those in rural or suburban regions.			
Bijnens (2022)	Multiple linear regression<br><br>Covariates: age, sex, education of mother, area deprivation index	Residential greenspace not associated with memory or visual processing speed.<br><br>Higher total greenspace and high green indices associated with shorter reaction time from Stroop and continuous performance test.<br><br>Access to small urban green and urban green associated with faster reaction on Stroop and continuous performance test.	No (NA)	NA	No
Binter (2022)	Linear regression (single exposure models)<br><br>Multi-exposure models<br><br>Covariates: area of inclusion/region; parent country of birth, residential area deprivation index,	Higher greenness/NDVI during pregnancy associated with higher verbal abilities.<br><br>Higher greenness/NDVI remained associated with verbal score in multi-exposure model.<br><br>No associations for distance	No (NA)	NA	No

	maternal age at recruitment, maternal education at recruitment, maternal pre-pregnancy body mass index (BMI), maternal smoking during pregnancy, paternal age at recruitment, paternal education at recruitment, paternal BMI at recruitment, season of birth, child age at assessment	to nearest major greenspace or non-verbal abilities.			
Brown (2018)	Multilevel logistic regression<br><br>Covariates: age, gender, racialized/ethnic group, and neighborhood income level	Reduced risk of AD diagnosis for Individuals living in Neighborhoods with higher greenness/NDVI values.	No (NA)	NA	No
Cerin (2021)	Generalized additive mixed models<br><br>Covariates: age, sex, English speaking background, education, Population density, percentage of commercial land use, land use mix, area-level socioeconomic disadvantage, residential self-selection related to recreational facilities	Percentage parkland associated with better memory and processing speed.	No (NA)	NA	No
Cherrie (2018)	Linear regression (examined accumulation and critical period).	Greater neighborhood parks in childhood and adulthood associated with slower cognitive decline later in life.	No (NA)	NA	No<br><br>(although employed life course models

	Covariates: sex, father's occupational social class (OSC), number of people per rooms in childhood household, childhood smoking status, adulthood OSC, alcohol consumption and adulthood smoking status.				to examine exposure as accumulation and critical periods)
Cherrie (2019)	Multilevel linear regression<br><br>Covariates: sex; father's occupational social class (OSC), number of people per rooms in childhood household, childhood smoking status, adulthood OSC, alcohol consumption and adulthood smoking status.	Park availability near home, school, and school commute during adolescence associated with les cognitive change (decline) in later life. No association between park availability at ages 4-11 and cognitive change in later life.	No (NA)	NA	No<br><br>(although used life course model framework)
Clarke (2012)	Multilevel linear regression<br><br>Covariates: age, gender, marital status, racialized/ethnic group, Employment status, socioeconomic position, index of comorbid conditions, social interaction, neighborhood socioeconomic disadvantage, neighborhood affluence, recreational	No association between park area and cognitive function.	No (NA)	NA	No

	centers, institutions, neighborhood disorder				
Crous-Bou (2020)	General linear models<br><br>Covariates: age, sex, education, body mass index (BMI), smoking at baseline, physical activity METs, diabetes status, self-report hypertension, self-reported high cholesterol, family history of AD before 75 years, apolipoprotein E (APOE) e4 status	No association between greenness/NDVI and cognition, episodic memory or executive function composites.<br><br>NDVI associated with greater AD regions of interest cortical thickness. No association between NDVI and hippocampal volume or ventricle volume.	No (NA)	NA	No
Dadvand (2015)	Linear mixed effects models<br><br>Covariates: age, sex, maternal education, residential neighborhood socioeconomic status	Positive association between total surrounding greenness index and 12-month enhancement in working memory and attention.<br><br>Greater commute route greenness associated with 12-month enhancement in working memory.<br><br>No other associations found.	No (NA)	NA	No
Dadvand (2017)	Negative binomial for count data and linear mixed models for continuous variables.<br><br>Covariates: age, sex, pre-term birth, maternal education, maternal cognitive performance, maternal smoking during pregnancy, and exposure to environmental tobacco	Greater neighborhood greenness (birth to 4-5 years old) associated with better attention at 4-5 years and greater greenness (birth to 7 years old) associated with better attention at 7 years old. Percentage of neighborhood woody vegetation >5m (VCF) not associated with attention.	No (NA)	NA	No

	smoke, neighborhood SES				
Dadvand (2018)	Separate regressions for each voxel in the brain using statistical parametric maps<br><br>Covariates: maternal education and individual socioeconomic status	Clusters in several (but not all) brain regions significantly associated with greenness/NDVI exposure since birth: left and right prefrontal cortex, left premotor cortex, and white matter.	No (NA)	NA	No
De Keijzer (2018)	Linear mixed effects models<br><br>Covariates: gender, ethnicity, education level, age, age squared, marital status, employment grade, neighborhood SES, diet, alcohol consumption, and smoking status.	Greater greenness/NDVI associated with slower decline over time in global cognition, reasoning and fluency, but not short term memory.	No (NA)	NA	No

Dockx (2022)	Multivariable linear regression<br><br>Covariates: child's age, sex, maternal education, child's average daily screen time, time of the examination	Association between greater greenspace and less pixel distance from the target of the Motor Screening Task.<br><br>Association between greater greenspace and less probability of error in Delayed Matching to Sample task.<br><br>Association between greater greenspace and greater percentage correct trials on Delayed Matching to Sample Tasks.	No (NA)	NA	No
Dzhambov (2019)	Linear regression models<br><br>Covariates: sex, age, education, city, population within 500m buffer, smoking, alcohol consumption, waist circumference, systolic blood pressure, total cholesterol, and blood glucose, NO2, road traffic noise	Greater greenness associated with better global cognition and verbal fluency. Greater greenness associated with greater cortical thickness in both hemispheres in the prefrontal cortex, bilateral fusiform gyrus, left precuneus and insula, and right cuneus. Greater greenness was not	No (NA)	NA	No

		associated with scores on the subtests of the CERAD-NB except the Verbal Fluency Test. No other associations observed.			
Falcon (2021)	General linear models in voxel basis<br><br>Covariates: age, sex, education, total intracranial volume (Sensitivity analysis also controlled for apolipoprotein (APOE) e4, smoking, body mass index (BMI), and physical activity)	Greater greenness/NDVI associated with greater grey matter volume in left middle frontal, precentral cortices, temporal pole.	No (NA)	NA	No
FangFang (2022)	Linear and logistic regression (sensitivity analysis using multilevel regression)<br><br>Covariates: age, sex, city, urban/rural, education, married, income, health behaviors (smoking, alcohol, sleep quality, physical activity), chronic diseases-hypertension and diabetes, mental status-depression	No associations between distance to park or percentage greenspace and MMSE score or mild cognitive impairment (MCI)	No (NA)	NA	No
Finlay (2021)	Gaussian generalized additive multilevel model (allowed for nonlinearity)<br><br>Covariates: age, years from baseline test, White racialized group, sex, education,	Greater number of parks associated with better cognitive test composite score.	No (NA)	NA	No

	neighborhood measures (owner occupied housing, proportion non-Hispanic Black, proportion below poverty)				
Flouri (2019)	Multilevel linear models<br><br>Covariates: age, gender, ethnicity, neighborhood deprivation, maternal education, family poverty, sports participation, computer gaming, and residential stability.	Greater % greenspace was associated with better spatial working memory.	No (NA)	NA	No
Hystad (2019)	Linear regression models<br><br>Covariates: Age, sex, household income, education, racialized group, marital status, years in current residence, city, population density.	Five-year decline in greenness/NDVI associated with worse reasoning.<br>Five-year average NDVI not associated with reaction time, reasoning, or working memory.<br>Five-year change in NDVI not associated with reaction time or working memory.	Yes (stratified results by racialized/ethnic group)	No difference in association between neighborhood greenness (NDVI) and cognitive functioning (reasoning, visual memory, and reaction time) between White and non-White participants.	No
Jimenez (2021)	Generalized additive models with natural splines with 3-4 knots<br><br>Covariates: sex, age, maternal IQ, maternal education, maternal race, paternal education, household annual income at enrolment in early pregnancy,	NDVI in early childhood associated with nonverbal IQ, verbal memory, visual motor abilities at mid-childhood.<br><br>No other associations between NDVI at any period and mid-childhood cognitive measures.<br><br>No associations between any NDVI measures and early-	No (NA)	NA	No

	neighborhood median annual income at birth, and greenness/NDVI exposure in all preceding age periods	childhood cognition measures.			
Jin (2021)	Logistic and linear regression<br><br>Covariates: age, gender, residency, education, occupation, married, smoking, drinking, diet, physical activity, activities of daily living, leisure activity score, and interview month	Higher quartiles of contemporaneous greenness/NDVI associated with lower odds of cognitive impairment. Increasing contemporaneous NDVI (continuous measure) associated with higher/better MMSE score.<br><br>No association between annual average NDVI and cognitive impairment or MMSE score.	No (NA)	NA	No
Ju (2021)	Logistic regression with backward elimination<br><br>Covariates considered: age, sex, education, income, job, married, area of residence, body mass index (BMI), healthy lifestyle, perceived stress, depressive symptoms, poor sleep	No association between perceived greenspace and subjective cognitive decline.	No (NA)	NA	No
Julvez (2021)	Multivariable linear regression to screen exposures. Then used deletion-substitution-addition algorithm to select exposures jointly associated with outcomes<br><br>Covariates: cohort, maternal education,	In single exposure model: Prenatal greenness/NDVI associated with greater fluid intelligence (CPM) and greater inattentiveness scores (ANT HRT-SE), but not associated with working memory (N-Back).<br><br>In multi-exposure model: Prenatal NDVI borderline	No (NA)	NA	No

	maternal age, child age, sex, trimester of conception (plus the other exposome measures for the multi-exposure model, mercury, alcohol intake, dimethyl thiophosphate, food intake, house crowding, environmental tobacco smoke, perfluorooctane sulfanate, Indoor $PM_{2.5}$)	associated with greater inattentiveness (p=0.08). No other associations between NDVI and other cognitive measures in multi-exposure model.			
Kuhn (2017)	Structural equation modeling<br><br>Covariates: wasteland, age, sex, years of education.	Greater amount of forest in neighborhood associated with greater amygdala integrity. No association between amount of forest and pACC or DLPFC integrity, or between amount of urban green and any brain measure.	No (NA)	NA	No
Lee (2021)	Multivariable linear mixed effects<br><br>Covariates (pregnancy period): mothers age, child's sex, mother's education, mother's IQ, exposure to environmental tobacco smoke (ETS), average NO_2 during prior 3 years, residential deprivation index, proportion of road density<br><br>Covariates (age 6): mother's age, child's sex, mother's education, mother's IQ,	Greater prenatal and postnatal built greenness associated with higher total IQ at age 6.<br><br>Prenatal and childhood built greenness associated with higher verbal IQ.<br><br>Table S4: Prenatal and childhood total greenness and built greenness associated with higher performance IQ.<br><br>No other associations (e.g., with natural greenness).	No (NA)	NA	No

	exposure to ETS at age 6, avg NO_2 during prior 3 years, household income, subjective noise level, distance to main road, physical activity duration at age 6, residential deprivation index, proportion of road density				
Lega (2021)	4-step mediation analysis (linear regression):<br>1) Association between greenness/NDVI and outcomes; 2) association between NDVI and stress as mediator; 3) association between stress and outcomes; 4) association between NDVI and outcomes through stress<br><br>Covariates: age, gender, education, index of multiple deprivation, frequency of visits to natural environment	NDVI was associated with better short-term memory (FDS) and better total memory (TDS), but not associated with working memory (BDS).	No (NA)	NA	No
Liao (2019)	Linear regression<br><br>Covariates: residence areas (rural, urban), household income, maternal age, maternal education, maternal pre-pregnancy body mass index (BMI), passive smoking during	Greater neighborhood greenness/NDVI at birth associated with better MDI scores.	No (NA)	NA	NA

	pregnancy, gestational age, and birth weight.				
Liu (2019)	Multilevel logistic regression<br><br>Covariates: occupation, insurance premium, number of comorbidities, hospital and clinics, urbanization status, other physical environment variables (playgrounds and sports venues), social environment (median annual fam income, illiterate age ≥65, and elderly living alone)	No association between parks, greenery, and square area and AD incidence.	No (NA)	NA	No
Liu (2020)	Multilevel logistic regression<br><br>Covariates: occupation, insurance premium, number of comorbidities, hospital and clinics, urbanization status, other physical environment variables (playgrounds and sports venues), social environment (median annual fam income, illiterate age ≥65, and elderly living alone)	No association between parks, greenery, and spare area and dementia incidence.	No (NA)	NA	No
Maes (2021)	Bayesian longitudinal multilevel models<br><br>Covariates: age, area deprivation, ethnicity, gender, parental	Adolescents cognitive development (executive function) improved with higher Daily Exposure Rate to natural space, greenspace (vs blue), and woodland.	No (NA)	NA	No

	occupation, school type, and air pollution				
Paul (2020)	Mixed effects Cox proportional hazards models<br><br>Covariates: age, sex, SES, comorbidities (coronary heart disease, diabetes, hypertension, congestive heart failure, stroke, arrhythmia, and traumatic brain injury), northern residence, population density, ambient air pollution (sensitivity analysis additionally adjusted for deprivation index and total count of fam doctors/ neurologists in census subdivision)	Greater greenness/NDVI associated with lower risk of incident dementia.	No (NA)	NA	No
Reuben (2019)	Full information maximum likelihood (FIML) estimated regression models (accounted for missing data) tested cross-sectional associations<br><br>Analysis of covariance model to test longitudinal associations (change in scores)<br><br>Covariates: sex, the child's educational-attainment polygenic score, family	Neighborhood greenness not associated with fluid ability, crystallized ability, executive function, attention, or working memory measured at any age.	No (NA)	NA	No

	socioeconomic background, and residential neighborhood socioeconomic status.				
Slawsky (2022)	Cox proportional hazards regression<br><br>Covariates: year, race, sex, treatment arm, recruitment site, neighborhood SES, education, activities of daily living mobility, mild cognitive impairment at baseline, body mass index (BMI), and apolipoprotein E (APOE) e4 status, rurality	In fully adjusted models, medium (but not high) greenspace associated with lower dementia risk (all cause, mixed/vascular, and Alzheimer's disease).	No (NA)	NA	No
Sylvers (2022)	Multilevel linear regression<br><br>Covariates: age, sex, racialized/ethnic group, education, walking minutes/day, physical activity level, physical health status, neighborhood SES, urban/rural, street connectivity, heavy traffic	Objective count of neighborhood parks not associated with overall cognition, but perceived lack of parks/playgrounds (somewhat serious or very serious) was associated with cognition.	No (NA)	NA	No
Tani (2021)	Multilevel Weibull survival models<br><br>Covariates: neighborhood features (sidewalk coverage, land slope, number of hospitals, grocery	No association between number of parks and dementia incidence.	No (NA)	NA	No

	stores, railway stations, bus stops, area of school district) and individual factors (age, sex, education, income, living situation, marital status, employment) and health (hypertension, diabetes, hearing loss, heart disease, stroke, depressive symptoms, Instrumental activities of daily living (IADL), cognitive complaints) and duration of residence				
Wang (2017)	Spearman's correlation coefficient (unadjusted)	No correlation between neighborhood greenness/NDVI and global cognition.	No (NA)	NA	No
Ward (2016)	Generalized linear mixed models<br><br>Covariates: sex, age, school	% time spent in greenspace not associated with any cognitive domain.	No (NA)	NA	No
Wu (2015)	Multilevel logistic regression<br><br>Covariates: age, gender, education, social class, chronic illness, and area deprivation.	Individuals living with highest quartile of neighborhood green space (versus lowest) had increased odds of cognitive impairment and dementia.	No (NA)	NA	No
Wu (2017)	Multilevel logistic regression<br><br>Covariates: age, gender, education, social class, chronic illness, and area deprivation.	Individuals living with highest quintile of neighborhood green space/private gardens (versus lowest) had increased odds of cognitive impairment. No associations between neighborhood green space and odds of dementia.	No (NA)	NA	No

Wu (2020)	Multilevel logistic regression<br><br>Covariates: age, education, gender, social class/assets, self-reported health, center/site	No association between % recreational green and % nature and odds of dementia<br><br>Greater distance to public park associated with lower odds of dementia.	No (NA)	NA	No
Wu (2021)	Zero inflated Poisson Models<br><br>Covariates were unclear, but appeared to include: PM2.5, percentage water, median annual household income, zip code, population density, and road density	Greater percentage greenspace associated with lower risk of AD diagnosis.	Yes (stratified models by racialized/ethnic group)	No difference in regression estimates between Black and White participants. Both demonstrated association between greater greenspace and lower risk of AD.	No
Yu (2018)	Multivariable regression path analysis<br><br>Covariates: age, sex, marital status, socioeconomic status, alcohol intake, diet quality, baseline frailty status	Greater neighborhood greenness not directly associated with cognition.	No (NA)	NA	No
Yuchi (2020)	Cox proportional hazard model (Parkinson's and non-AD outcomes)<br><br>Conditional logistic regression for AD outcome<br><br>Covariates: neighborhood characteristics	Greater neighborhood greenness/NDVI associated with lower hazard ratio for non-Alzheimer's disease and Parkinson's disease. Greater neighborhood greenness associated with increased odds of Alzheimer's disease.	No (NA)	NA	No

	(household income, education, ethnicity), age, sex, and comorbidity.				
Zhu (2019)	Linear regression, logistic regression, linear mixed effects regression, logistic mixed effect regression<br><br>Covariates: age, gender, ethnicity, married, geographic region, urban/rural residence, education, occupation, financial support, social and leisure activity, smoking status, alcohol consumption, and physical activity	Greater greenness/NDVI associated with higher (better) MMSE score and lower odds of cognitive impairment (MMSE<24) in serial cross-sectional analyses.<br><br>Greater NDVI associated with higher (better) MMSE scores but not cognitive impairment (MMSE<24) in longitudinal analyses.<br><br>Individuals living in places where NDVI decreased over time had greater odds of decrease in MMSE score over time.	No (NA)	NA	No
Zhu (2020)	Logistic regression with generalized estimating equations<br><br>Covariates: age, sex, marital status, urban/rural, education, financial support, occupation, smoking/alcohol habits, social and leisure activity.	Individuals living in highest quartile of neighborhood greenness/NDVI had lower odds of cognitive impairment.	No (NA)	NA	No
Zijlema (2017)	Linear and logistic multilevel models<br><br>Covariates: age, sex, education,	Greater residential distance to natural outdoor environments associated with greater cognitive test completion time. Residential	No (NA)	NA	No

	neighborhood socioeconomic status, time spent away from home, and Color Trails Test (CTT) quality	greenness, percentage residential natural environment, self-reported natural environment visits, and self-reported time spent visiting natural environment not associated with cognition.			
